# Supplementary material for: Type III ATP synthase is a symmetry-deviated dimer that induces membrane curvature through tetramerization
Source: Nat Commun. 2020 Oct 22;11:5342. doi: 10.1038/s41467-020-18993-6 (PMC7583250; doi:10.1038/s41467-020-18993-6)
Supplement: Supplementary file 1 — Supplementary Information [file 41467_2020_18993_MOESM1_ESM.pdf]

Supplementary Information

Type III ATP synthase is a symmetry-deviated dimer that induces membrane curvature through tetramerization

Rasmus Kock Flygaard[1,2†], Alexander Mühleip[1,2†], Victor Tobiasson[1,2] and Alexey Amunts[1,2*]

[1] Science for Life Laboratory, Department of Biochemistry and Biophysics, Stockholm University, 17165 Solna, Sweden.
[2] Department of Medical Biochemistry and Biophysics, Karolinska Institute, 17177 Stockholm, Sweden.

† Both authors contributed equally to this work.

* email: amunts@scilifelab.se

This file includes:

Supplementary Fig. 1 to 14
Supplementary Tables 1 to 2

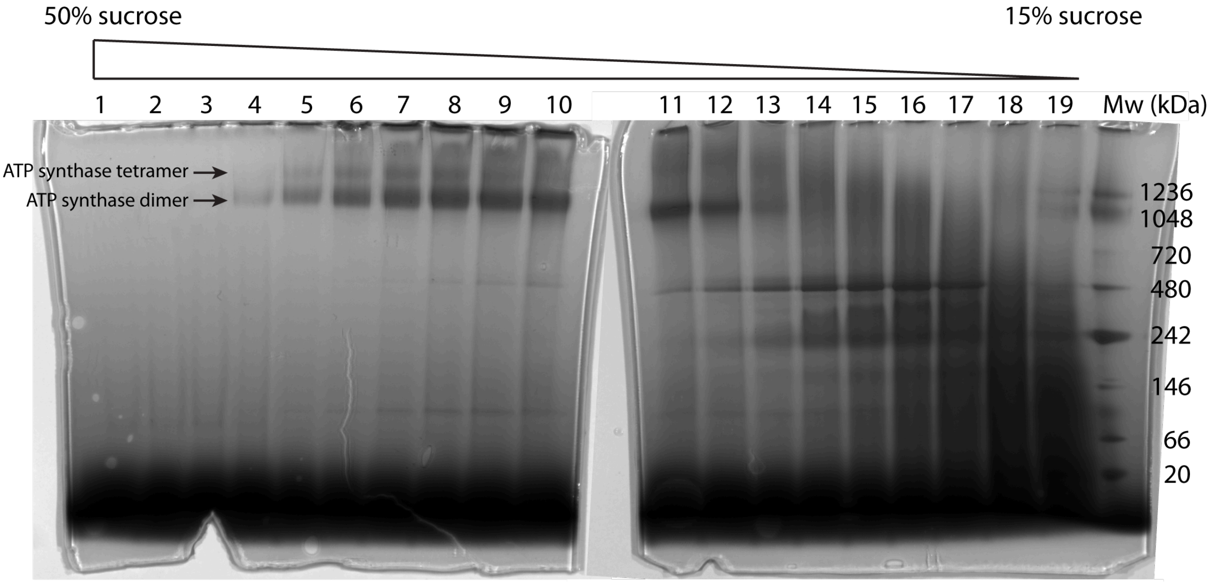

Supplementary Fig. 1 | Digitonin-solubilized complexes analyzed with BN-PAGE. Cleared digitonin-solubilized mitochondrial lysate was sedimented through 15-50% w/v sucrose gradients and all fractions (1-19) analyzed on blue-native PAGE gradient gels. Molecular weight (Mw) markers are shown in the right-most lane on the second gel. Fractions 5-8 (~35-40% w/v sucrose) show two bands, a major band (ATP synthase dimer) and a minor band a (ATP synthase tetramer) indicating successful solubilization of ATP synthase dimers and higher oligomers.

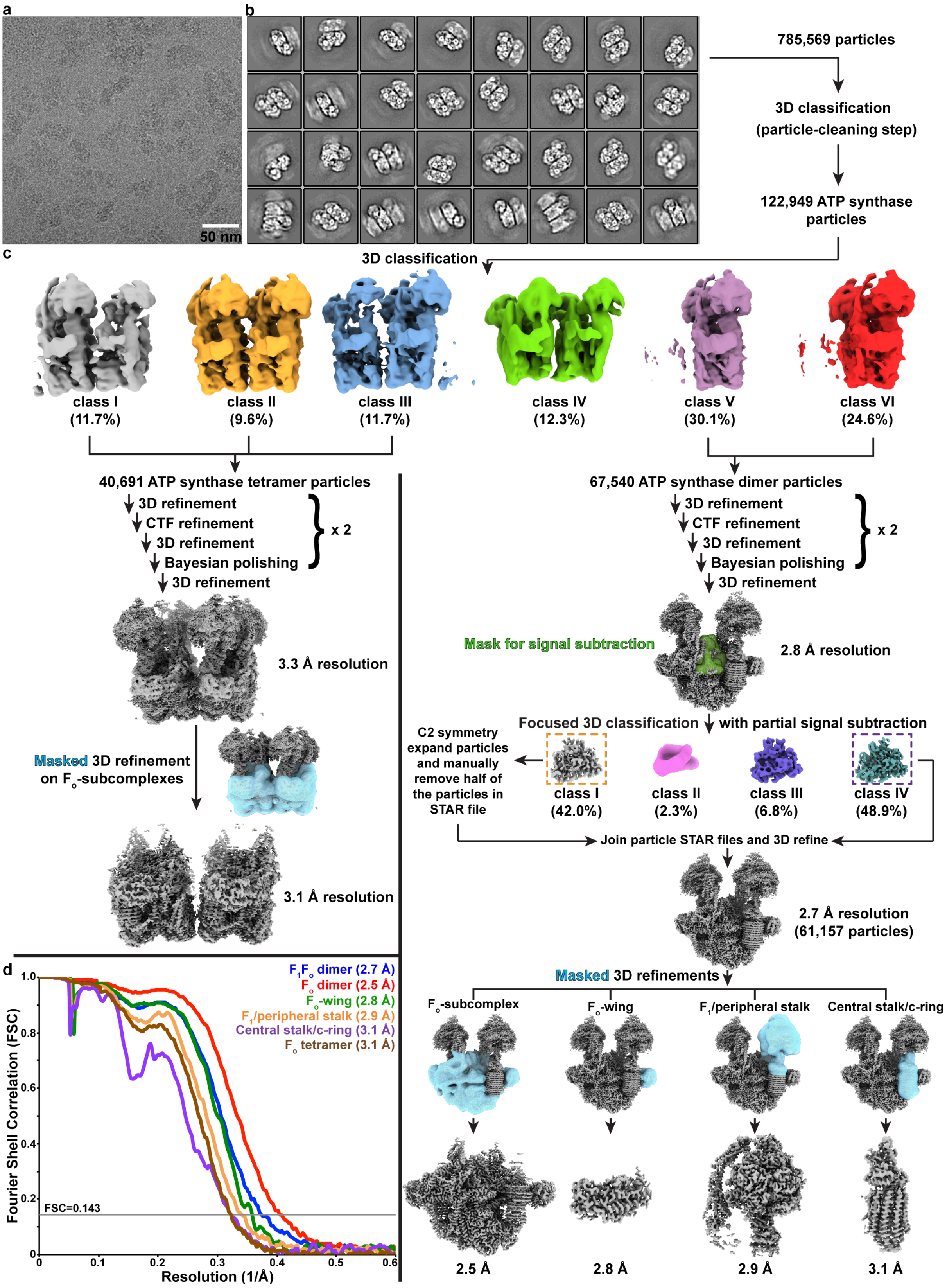

Supplementary Fig. 2 | Cryo-EM data processing. a, representative motion-corrected micrograph of ATP synthase particles. **b,** 2D class averages of ATP synthase particles. **c,** particle processing workflow in RELION-3.1. Left: image processing scheme of ATP synthase tetramer particles, right: processing of ATP synthase dimer particles. **d,** Fourier shell correlation from RELION postprocessing step of the final six reconstructions.

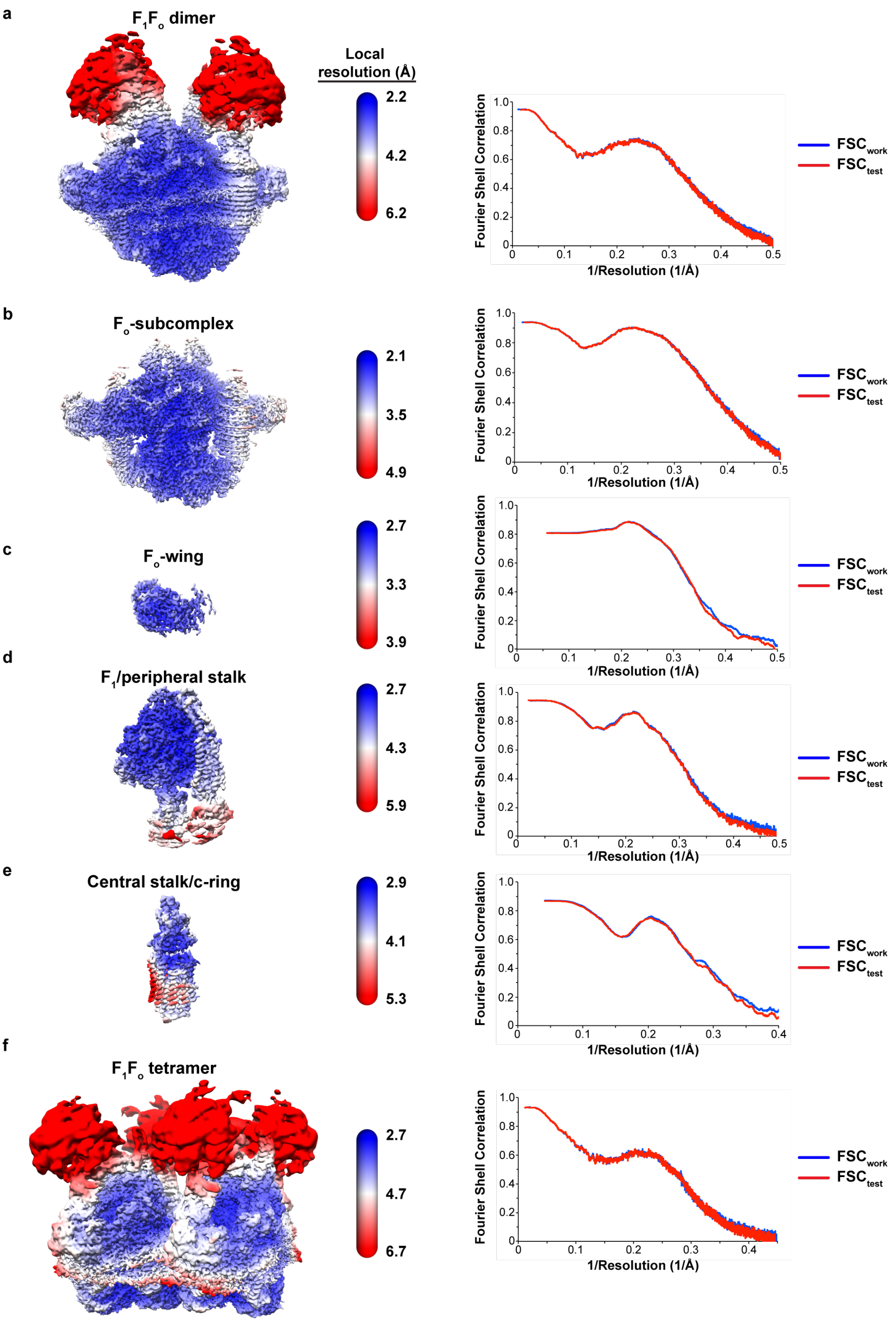

Supplementary Fig. 3 | Local resolution of final reconstructions and map-model validation. Left column shows the final reconstructions colored according to local resolution estimated using local resolution calculation in RELION. Right column shows Fourier shell correlations FSC_{work} and FSC_{test} for validation of models built and refined into the respective maps. **a,** F_1F_o dimer reconstruction. **b,** F_o dimer reconstruction. **c,** F_o-wing reconstruction. **d,** F_1/peripheral stalk reconstruction. **e,** central stalk/c-ring reconstruction. **f,** F_o tetramer reconstruction.

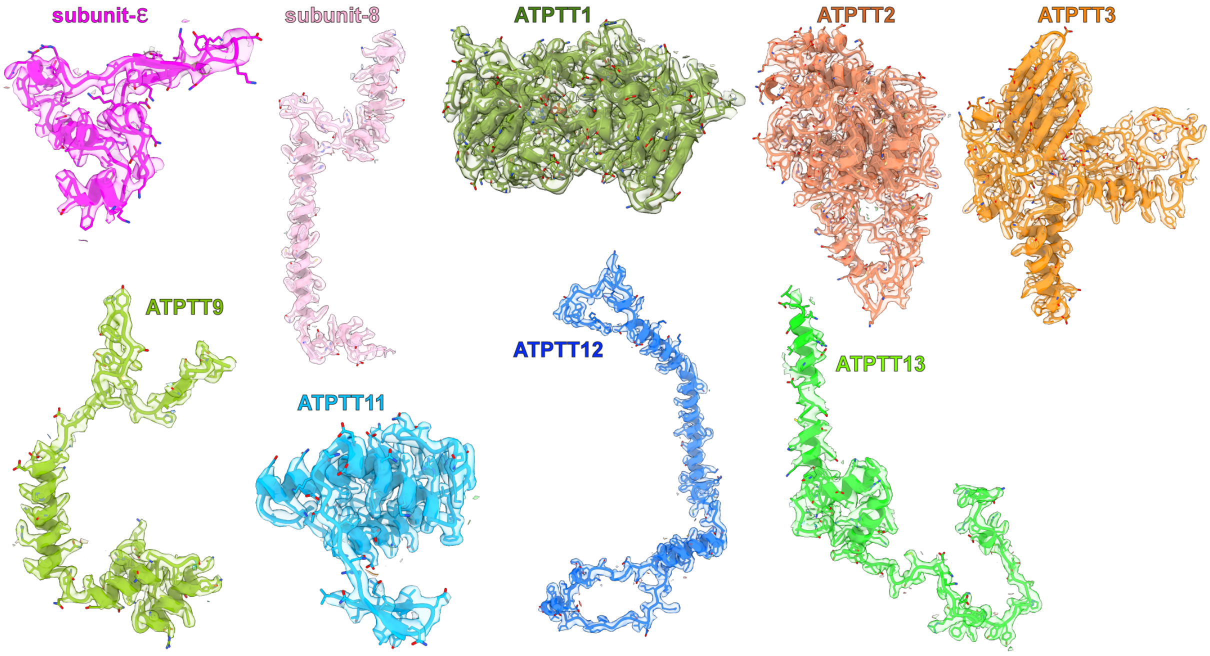

Supplementary Fig. 4 | Newly discovered subunits in type III ATP synthase. Subunits identified directly from the cryo-EM map are shown with local-resolution filtered density carved 2 Å around the model atoms.

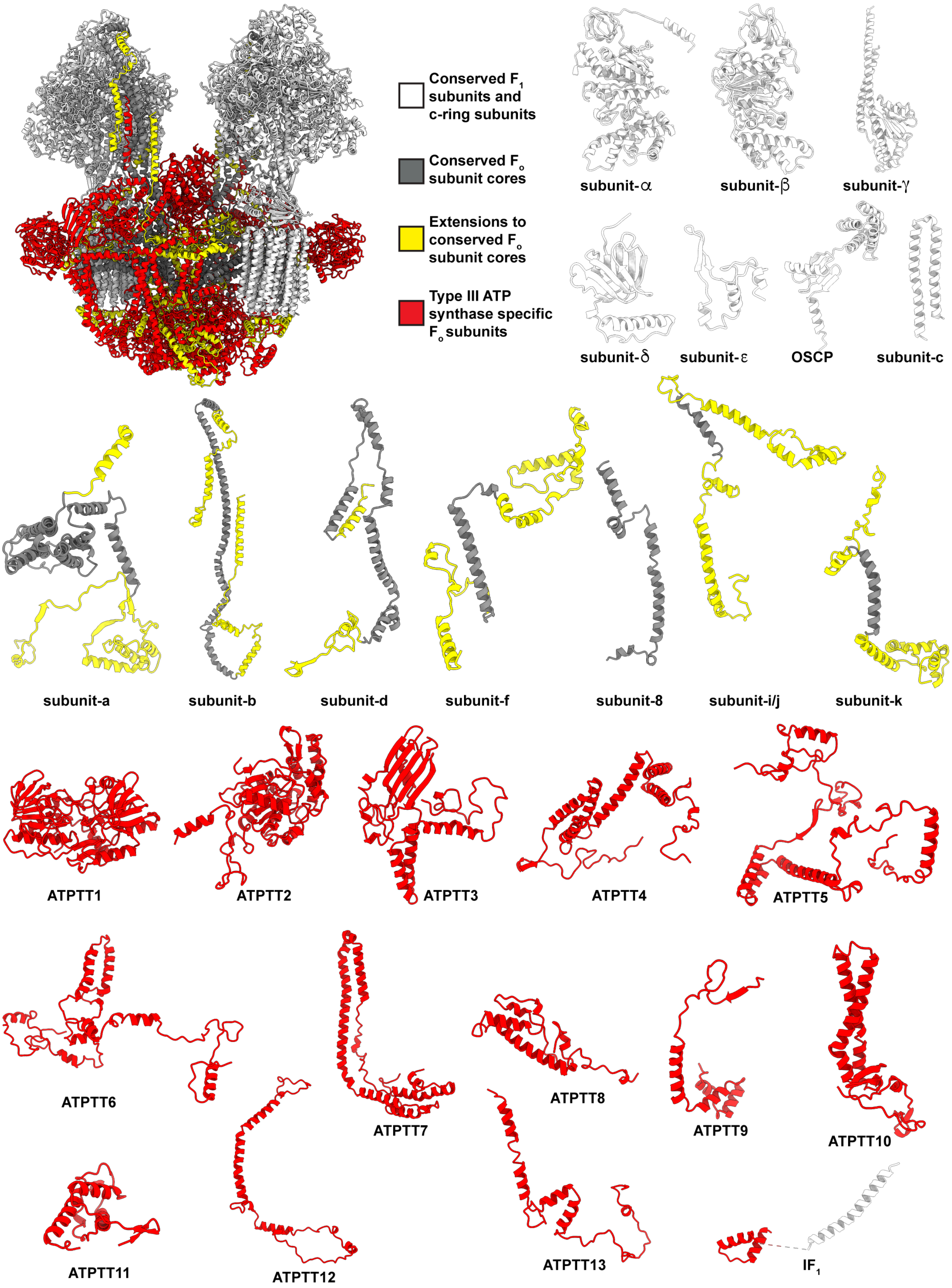

Supplementary Fig. 5 | Individual subunits of the type III ATP synthase. Tertiary folds of all the built proteins are shown. Conserved structures and core domains of F_1 and F_o subunits are shown in white and grey, respectively. Extensions to F_o subunit cores are yellow and type III specific proteins are red.

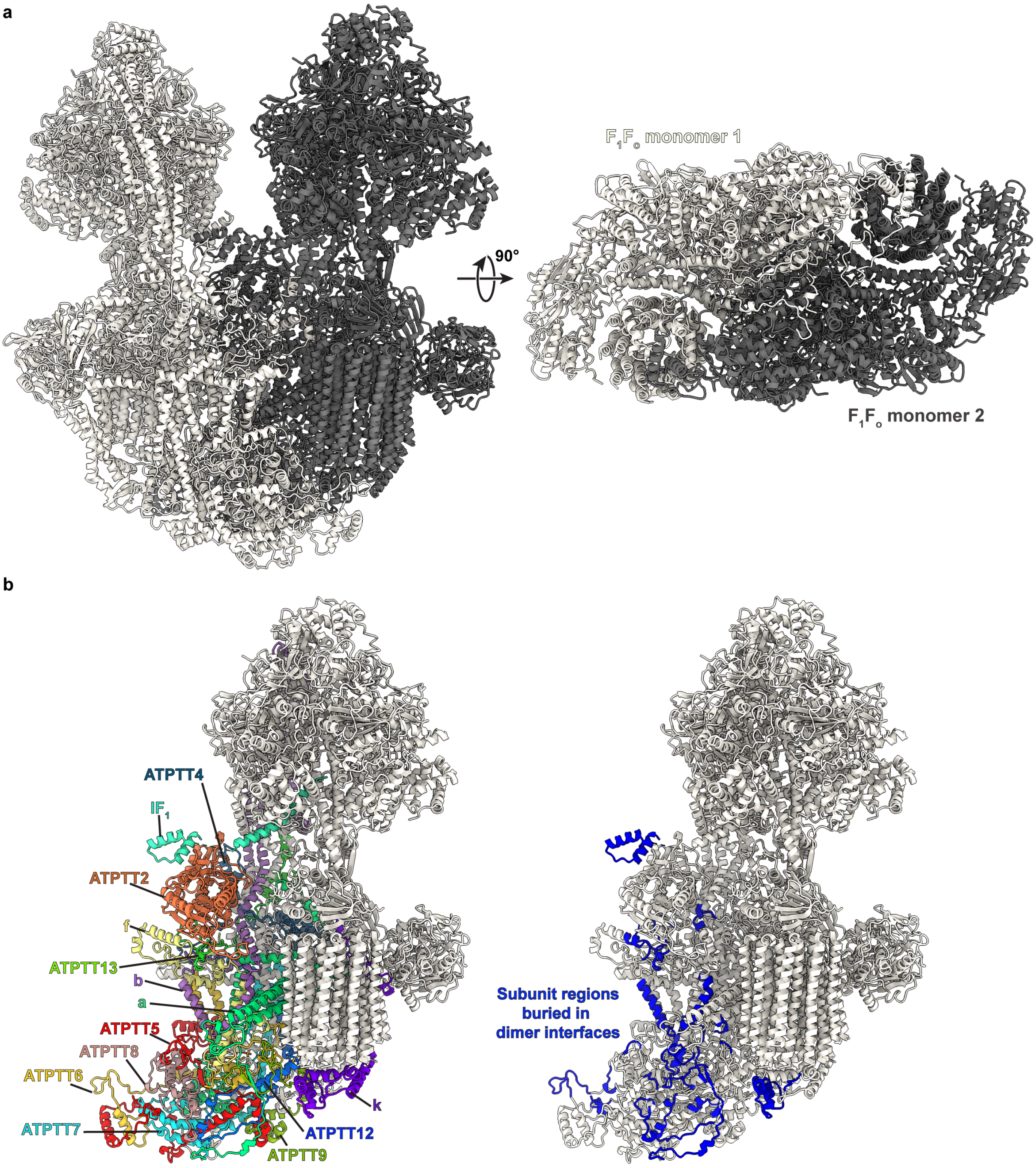

Supplementary Fig. 6 | Dimerization of the type III ATP synthase. a, two monomers are shown in white and grey, illustrating that the extensive dimerization occurs in the membrane, matrix, and lumen, providing the largest dimer interface reported for mitochondrial ATP synthase. **b,** View from the interface, left panel: F_o subunits in one monomer involved in dimer interface interactions are indicated; right panel: secondary structure elements forming direct contacts between the monomers are shown in blue.

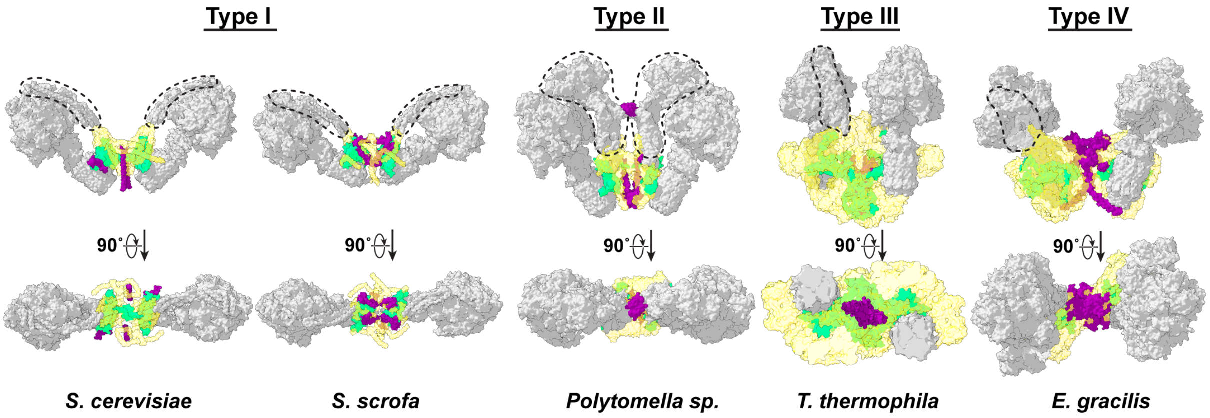

Supplementary Fig. 7 | Comparison of dimerization modes between mitochondrial ATP synthases. F_1/c-ring and peripheral stalk are gray, F_o subcomplex is transparent gold, subunit-*a* is green, F_o subunit domains forming dimer interface are magenta. The peripheral stalks are encircled by dashed lines for orientation. In type III and type IV ATP synthase, the laterally offset peripheral stalks allows just one peripheral stalk to be seen. The following models were used: type I ATP synthase - *Saccharomyces cerevisiae* (PDB 6B8H) [https://www.rcsb.org/structure/6B8H], *Sus scrofa* (PDB 6J5K) [https://www.rcsb.org/structure/6J5K]; type II ATP synthase - *Polytomella sp.* (PDB 6RD4) [https://www.rcsb.org/structure/6RD4]; type III ATP synthase - current work; type IV ATP synthase *Euglena gracilis* (PDB 6TDU) [https://www.rcsb.org/structure/6TDU].

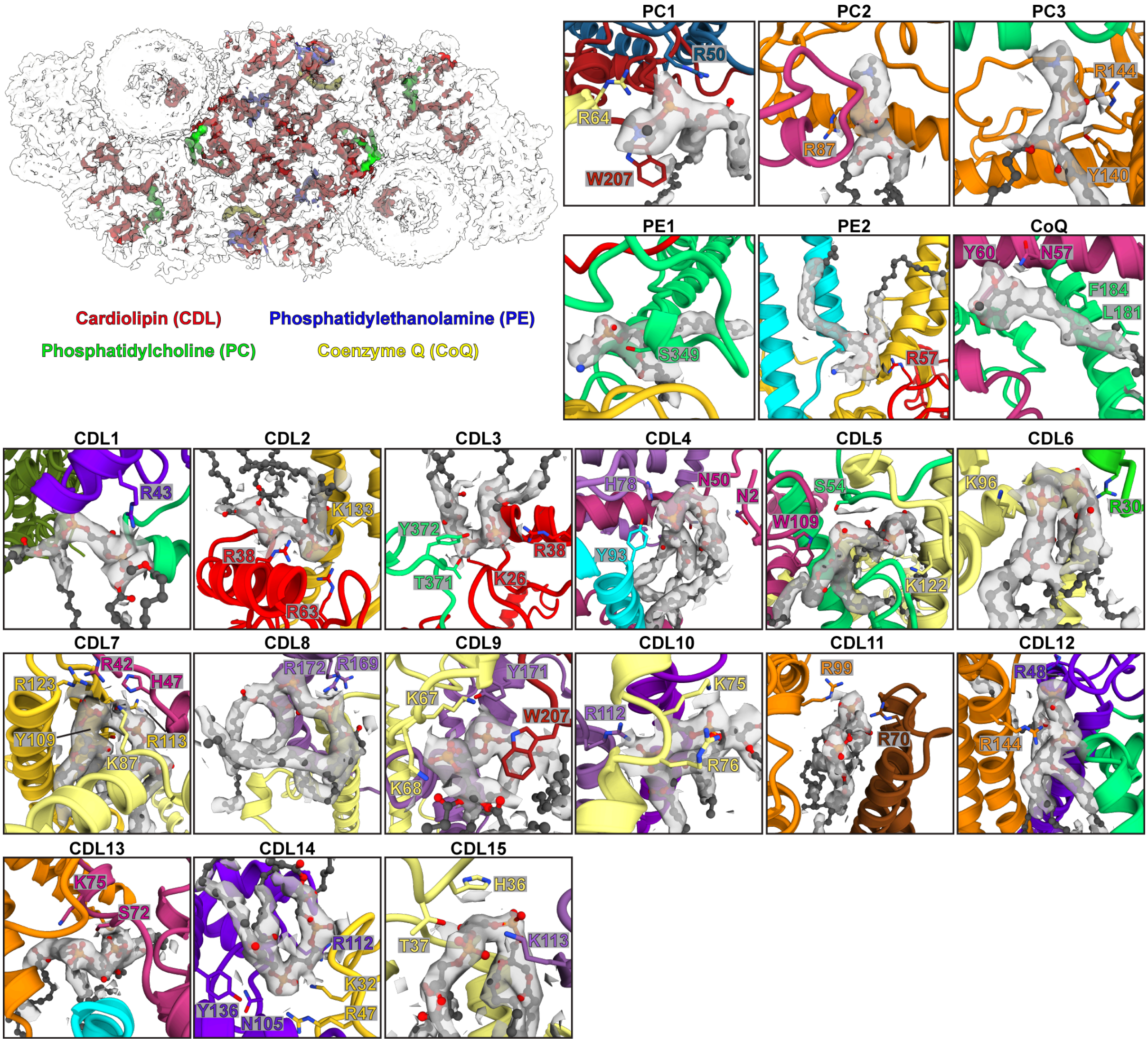

Supplementary Fig. 8 | Binding sites for lipids and coenzyme Q in the ATP synthase dimer structure. Overview of the density features of 30 cardiolipins, 6 phosphatidylcholines, 4 phosphatidylethanolamines, 2 CoQ molecules. All lipid/CoQ binding sites are C_2-symmetry related around the pseudo-C_2 symmetry axis of the F_o-subcomplex. Close-up views include density carved 2.6 Å around the lipid/CoQ molecule.

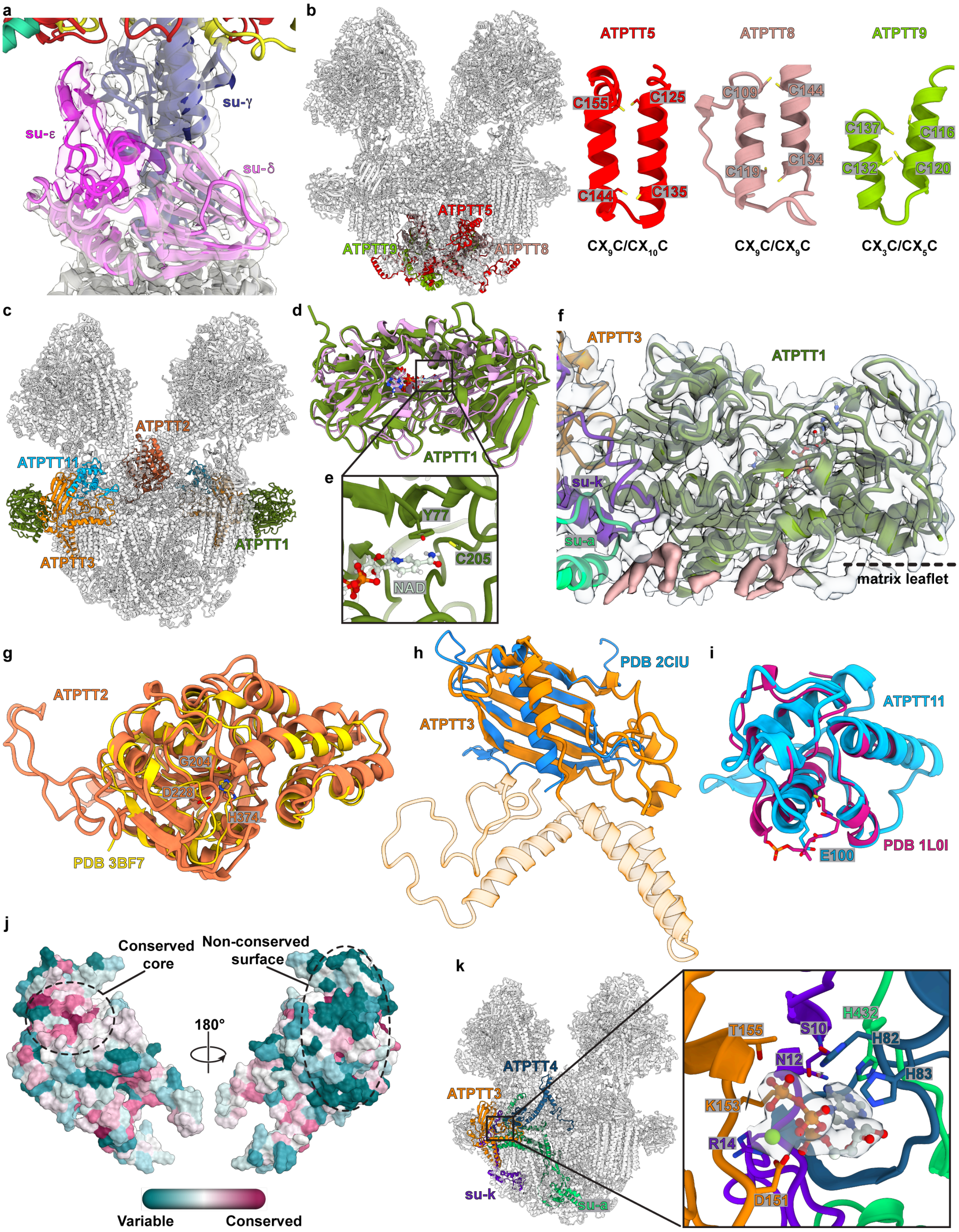

Supplementary Fig. 9 | Features of newly identified subunits. a, central stalk region highlighting density that allowed model building subunit-ε (magenta). **b,** locations and structures of ATPTT5 (red), ATPTT8 (light brown) and ATPTT9 (light green), with the identified CHCH-domains. **c,** overview showing location of matrix-side F_o subunits with conspicuous globular tertiary folds. **d,** ATPTT1 (green) superposed with the structure of human sulfide:quinone oxidoreductase (pink) (PDB 6MP5)

[https://www.rcsb.org/structure/6MP5]. **e,** nicotinamide group of NAD. **f,** ATPTT1 locates close to the matrix leaflet of the inner mitochondrial membrane. Protein density of the F_o-wing map is shown as transparent surface with lipid/detergent density features colored light brown. **g,** ATPTT2 (orange-brown) superposed with the structure of *Escherichia coli* α/β-hydrolase protein ybfF (yellow) (PDB 3BF7) [https://www.rcsb.org/structure/3BF7]. ATPTT2 contains an incomplete Ser-His-Asp catalytic triad with the Ser-residue mutated to Gly204. **h,** ATPTT3 contains a C-terminal fold (orange), which superposes with yeast TIM21 IMS-domain (blue) (PDB 2CIU) [https://www.rcsb.org/structure/2CIU]. **i,** ATPTT11 adopts a core fold of four helices superposing well with *E. coli* acyl-carrier protein (dark pink) (PDB 1L0I) [https://www.rcsb.org/structure/1L0I]. ATPTT11 contains a Glu100 residue instead of a serine, which normally conjugates with a phosphopantetheine group. **j,** ATPTT2 colored by sequence-conservation calculated using ConSurf. Residues at the core display a high degree of conservation whereas the surface residues are more variable. **k,** F_o-bound Mg-ATP ligand.

a

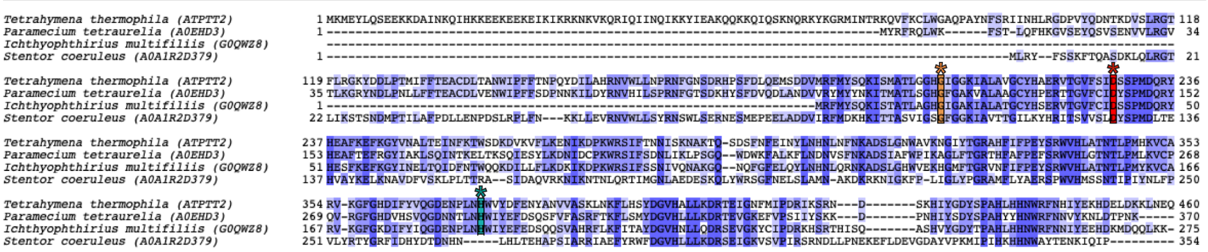

b

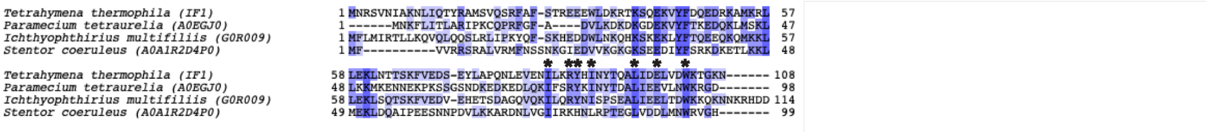

Supplementary Fig. 10 | Sequence alignments of ciliate ATPTT2 and IF$_1$. a, Clustal-Omega sequence alignment of *T. thermophila* ATPTT2 sequence with corresponding candidate sequences in other ciliate organisms (left side). UniProt entries for ATPTT2-candidate sequences are shown in brackets. Conserved residues of the inactive catalytic triad are shown in colored boxes marked by asterisks. **b,** Clustal-Omega sequence alignment of *T. thermophila* IF$_1$ sequence with corresponding candidate sequences in other ciliate organisms (left side). UniProt entries for IF$_1$-candidate sequences are shown in brackets. Conserved residues in the C-terminal region important for dimerization are marked by black asterisks.

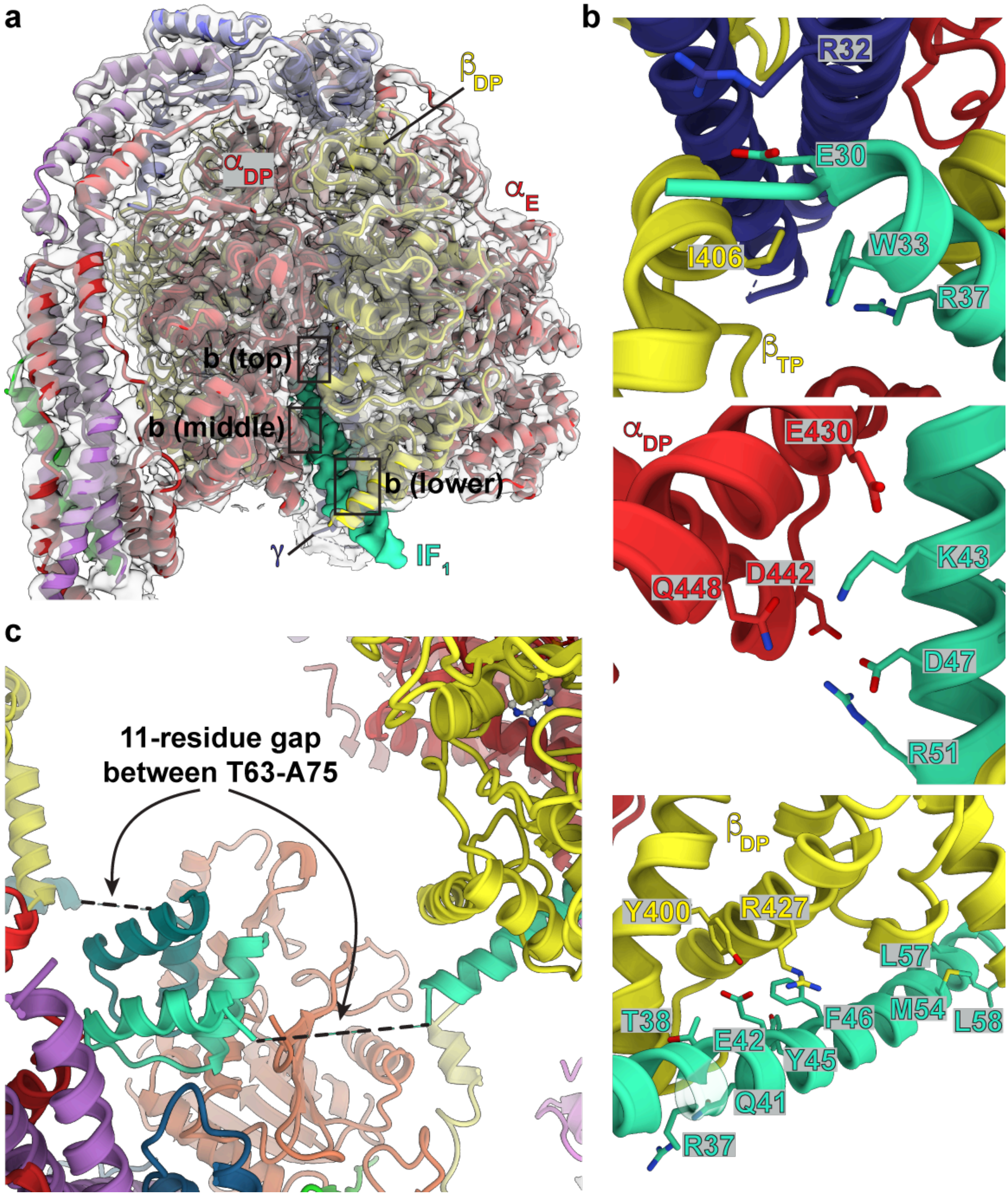

Supplementary Fig. 11 | IF_1 is bound to F_1- and F_o-subcomplexes. a, F_1/peripheral stalk model in colors and local resolution-filtered cryo-EM map in transparent gray. IF_1 density colored green-cyan is observed bound to the β_{DP} subunit extending to the γ subunit (dark blue). Windowed regions indicated the regions for close-up views shown in b panels. **b,** IF_1 interactions in the F_1-subcomplex to subunit γ and subunit β_{TP} (top panel), subunit α_{DP} (middle panel) and the extensive interface with subunit β_{DP} (lower panel). **c,** a gap of 11 residues was not resolved in the cryo-EM density maps between the IF_1 N-terminal helix projecting from the F_1-subcomplex and the F_o-bound C-terminal homodimer.

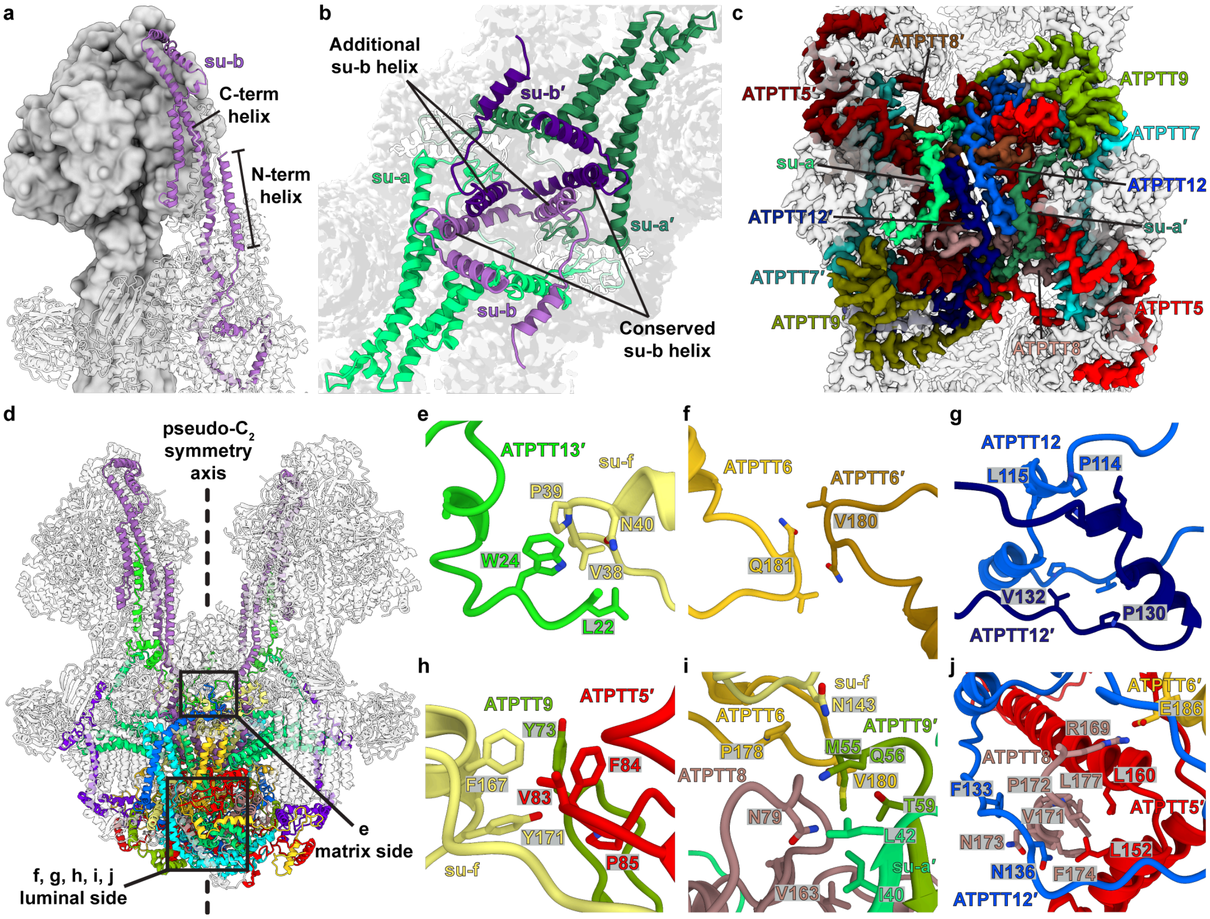

Supplementary Fig. 12 | Extensions of subunit-*a* and -*b* and dimer interfaces. a, subunit-*b* expands the peripheral stalk structure through an extended N-terminal helix, which together with the canonical C-terminal helix comprises the major part of the peripheral stalk. **b,** matrix view showing how subunit-*a* interacts with the canonical helix of conserved subunit-*b* as well as with a non-canonical helix of subunit-*b*′. **c,** luminal view showing the N-terminal extensions of both subunit-*a* copies tying together the F_o-subcomplex by interacting with symmetry-related F_o subunits. Dashed white line indicates the border between symmetry related monomers. **d,** overview of ATP synthase dimer highlighting F_o subunits involved in dimer interfaces. Windows show locations of panel e-j close-up views. **e,** matrix-side dimer interface between subunit-*f* and ATPTT13′. **f,g,** dimer interface between symmetry-related ATPTT6, ATPTT12. **h-j,** dimer interface between symmetry-unrelated subunits.

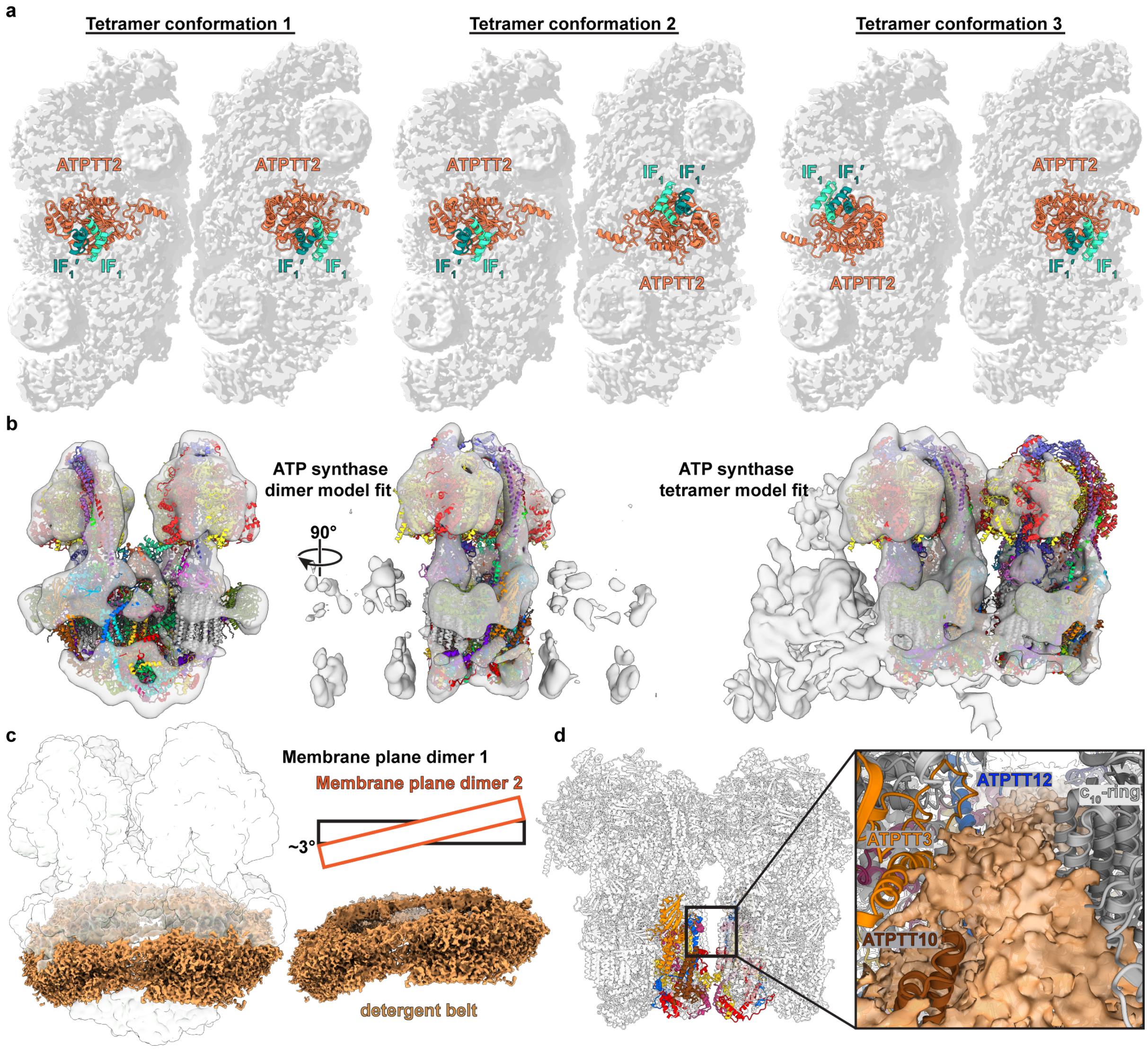

Supplementary Fig. 13 | Multiple conformations of type III ATP synthase tetramer. **a,** matrix view of the type III ATP synthase tetramer in three conformations brought about by the pseudo-symmetric nature of the dimer. F_o subunit ATPTT2 and IF_1 C-terminal dimer are shown in colored ribbons. **b,** ATP synthase dimer and tetramer atomic models fitted into subtomogram average (EMD-3441) [https://www.ebi.ac.uk/pdbe/entry/emdb/EMD-3441]. The tetramer model was fitted with one dimer copy to the density map, showing the second dimer copy in the model to agree with density of a second dimer in the reconstruction. **c,** end-view of ATP synthase tetramer (transparent light grey) with detergent/membrane density (light brown). Isolated detergent/membrane density shows the curvature induced by the ATP synthase tetramer, ~3° between dimer membrane planes **d,** lipid-mediated tetramer interface between F_o subunits ATPTT3, ATPTT10 and ATPTT12 in one dimer copy and the c-ring in the second dimer copy. Unmodeled lipid density is colored in light brown.

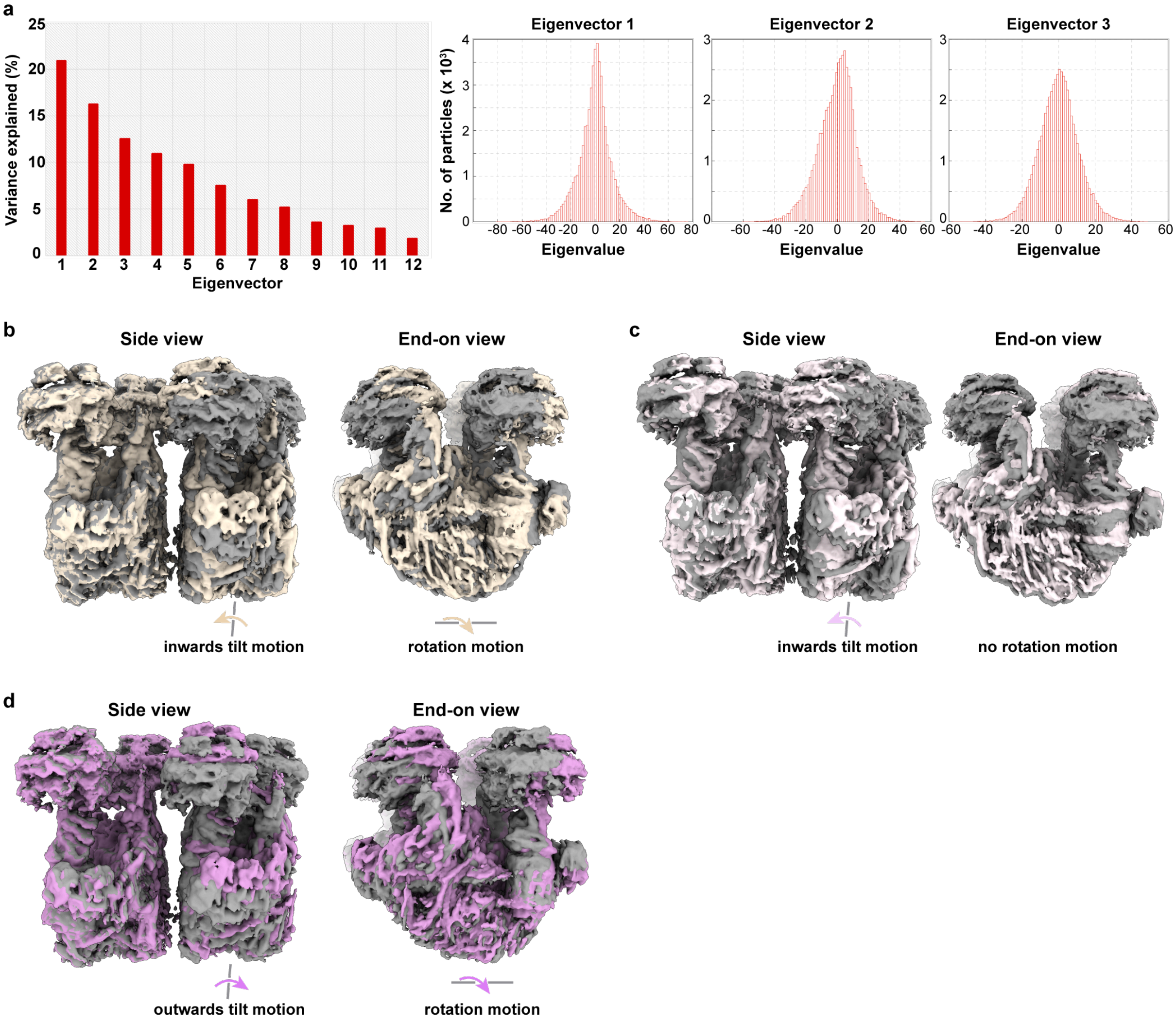

Supplementary Fig. 14 | Multi-body refinement analysis. a, left-side panel shows the multi-body refinement output from RELION-3.1 of the variance explained by eigenvectors. The first three eigenvectors together explain approximately 49% of the motion or flexibility in the ATP synthase tetramer particles. The last 9 eigenvectors account for the remaining ~51% of motion, making the interpretation non-trivial. Right-side panels show histograms of the eigenvalue distributions of the first three eigenvector components. The mono-model distributions indicate continuous motions with no distinct states. **b-d,** side and end-on views of reconstructions showing the motion along eigenvector 1 (b), eigenvector 2 (c) and eigenvector 3 (d) with start (grey) and end (colors) as the two extremes. As clearly seen in the side views, reconstructions are aligned on dimer to highlight the motion of one dimer with respect to the other. **b,** the motion along eigenvector 1 is a combination of an inwards tilt motion together with a rotation motion. **c,** the motion along eigenvector 2 consists mainly of an inward tilt motion with no obvious rotation motion. **d,** the motion along eigenvector 3 is a combination of one dimer tilting outwards while also rotating.

Supplementary Table 1

Data collection	F_1F_o dimer	F_o dimer	F_o-wing	F_1/peripheral stalk	c-ring/central stalk	F_o tetramer
**Microscope**	Titan Krios					
**Voltage (kV)**	300					
**Camera**	K2 Summit					
**Magnification**	165,000					
**Exposure (e⁻/Å²)**	30.9					
**Pixel size (Å)**	0.83					
**Defocus range (µm)**	0.6-2.6					
**Movies collected**	15439					
**Frames / movie**	20					
**Data processing**						
**Initial particles**	122,949					122,949
**Final particles**	61,157					40,691
**Symmetry**	C1	C1	C1	C1	C1	C2
**Map resolution (Å)**	2.7	2.5	2.8	2.9	3.1	3.1
**- FSC threshold**	0.143	0.143	0.143	0.143	0.143	0.143
**B-factor sharpen**	-12.6	-12.5	-18.5	-26.2	-54.2	-53.0
**EMD-ID**	10860	10859	10857	10862	10858	10861
**Model refinement statistics**						
**CC (map/model)**	0.72	0.88	0.88	0.87	0.82	0.64
**Resolution (map/model)**	2.59	2.53	2.93	3.05	3.43	3.39
**- FSC threshold**	0.5	0.5	0.5	0.5	0.5	0.5
**Atoms (not H)**	142054	69840	3439	27681	8955	284108
**Residues**	17337	7929	417	3598	1176	34674
**Lipids/CoQ**	40/2	40/2	-	-	-	80/4
**ATP/ADP/NAD**	8/4/2	2/-/2	-/-/1	3/2/-	-	16/8/4
**Mg-ions**	12	2	-	5	-	24
**Model B-factor (Å²)**						
- **Protein**	76.03	59.29	76.61	84.04	115.19	76.03
- **Lipids**	65.27	64.22	-	-	-	65.27
- **Ligands**	65.27	64.22	76.76	67.32	-	65.27
**Rotamer outliers (%)**	0.28	0.30	1.67	0.27	0.63	0.28
**Ramachandran (%)**						
- **Outliers**	0.01	0.00	0.00	0.00	0.00	0.01
- **Allowed**	2.15	2.29	6.02	3.67	2.26	2.16
- **Favoured**	97.84	97.71	93.98	96.33	97.74	97.83
**Clash score**	2.26	1.57	3.70	4.85	6.58	2.36
**MolProbity score**	1.04	0.97	1.73	1.50	1.42	1.05
**RMSD**						
- **Bonds (Å)**	0.003	0.005	0.008	0.005	0.004	0.002
- **Angles (°)**	0.500	0.600	0.695	0.568	0.664	0.483
**EMRinger score**	4.21	5.88	5.38	3.79	2.33	2.92
**PDB ID**	6YNY	6YNX	6YNV	6YO0	6YNW	6YNZ

Supplementary Table 2

Subunit name	Gene name	UniProt ID	Residues in total	Residues built	Location of subunits*	Comments
**F_1 subcomplex:**						
α	TTHERM_00571860	Q24HY8	546	32-545	MA	
β	TTHERM_00585260	I7LZV1	497	27-496	MA	
γ	TTHERM_00118610	Q22Z05	299	24-298	MA	
δ	TTHERM_01094890	Q22ZH1	158	24-157	MA	
ε	TTHERM_00682930	I7MMW3	71	2-69	MA	
OSCP	TTHERM_00684790	I7MMI7	219	32-219	MA	
IF_1	TTHERM_00145490	I7M7C0	108	28-63 + 75-106	MA	
**F_o subcomplex:**						
a	ymf66	Q951C1	446	14-446	MA+ME+CL	
b	TTHERM_00127260	I7MJ84	381	27-380	MA+ME	
c	atp9	Q951A5	76	1-75	ME	
d	TTHERM_01188360	Q239R1	234	29-234	MA+ME	
f	TTHERM_00571670	Q24I07	204	2-201	MA+ME+CL	
i/j	TTHERM_00529760	I7LZW2	209	1-209	MA+ME+CL	
k	TTHERM_00823660	I7LSX6	179	1-179	MA+ME+CL	
8	ymf56	Q950Y8	100	4-99	MA+ME	
ATPTT1	TTHERM_00137990	I7LVQ8	480	27-443	MA	Rossman fold
ATPTT2	TTHERM_00145440	I7M7B9	460	94-458	MA	α/β hydrolase fold
ATPTT3	TTHERM_00136220	I7M8Q3	286	31-286	MA+ME	TIM21 IMS fold
ATPTT4	TTHERM_00486310	I7MCZ0	268	38-268	MA	
ATPTT5	TTHERM_01528510	Q228N4	273	5-273	CL	CHCHD fold
ATPTT6	TTHERM_00649060	I7MCQ6	247	2-247	ME+CL	
ATPTT7	TTHERM_00316290	I7M980	221	1-221	ME+CL	
ATPTT8	TTHERM_00289380	I7LVK6	179	61-179	CL	CHCHD fold
ATPTT9	TTHERM_01001220	Q24HK1	154	55-153	CL	CHCHD fold
ATPTT10	TTHERM_00825290	I7LZE5	152	2-151	ME+CL	
ATPTT11	TTHERM_00927010	Q22DV8	152	45-152	MA	Acyl-carrier fold
ATPTT12	TTHERM_00068120	I7M0G0	149	5-149	MA+ME+CL	
ATPTT13	TTHERM_00444800	I7MLU7	145	22-145	MA	

*MA=matrix, ME=membrane, CL=crista lumen
