# Supplementary material for: Type III ATP synthase is a symmetry-deviated dimer that induces membrane curvature through tetramerization
Source: Nat Commun. 2020 Oct 22;11:5342. doi: 10.1038/s41467-020-18993-6 (PMC7583250; doi:10.1038/s41467-020-18993-6)
Supplement: Supplementary file 4 — Description of Additional Supplementary Files [file 41467_2020_18993_MOESM4_ESM.docx]

Description of Additional Supplementary Files

Title: Supplementary Video 1

Description: Overview of the type III ATP synthase structure
